# Detection of Salivary miRNAs Reflecting Chronic Periodontitis: A Pilot Study

**DOI:** 10.3390/molecules24061034

**Published:** 2019-03-15

**Authors:** Kohei Fujimori, Toshiki Yoneda, Takaaki Tomofuji, Daisuke Ekuni, Tetsuji Azuma, Takayuki Maruyama, Hirofumi Mizuno, Yoshio Sugiura, Manabu Morita

**Affiliations:** 1 Department of Preventive Dentistry, Okayama University Graduate School of Medicine, Dentistry and Pharmaceutical Sciences, 2-5-1 Shikata-cho, Kita-ku, Okayama 700-8558, Japan; kfujimori@s.okayama-u.ac.jp (K.F.); dekuni7@md.okayama-u.ac.jp (D.E.); pc2o46pj@okayama-u.ac.jp (H.M.); de421022@s.okayama-u.ac.jp (Y.S.); mmorita@md.okayama-u.ac.jp (M.M.); 2 Department of Community Oral Health, Asahi University School of Dentistry, 1851-1 Hozumi, Mizuho, Gifu 501-0296, Japan; tomofu@dent.asahi-u.ac.jp (T.T.); tetsuji@dent.asahi-u.ac.jp (T.A.); 3 Center for Innovative Clinical Medicine, Okayama University Hospital, 2-5-1 Shikata-cho, Kita-ku, Okayama 700-8558, Japan; t-maru@md.okayama-u.ac.jp

**Keywords:** saliva, periodontitis, microRNA

## Abstract

The purpose of this cross-sectional pilot study was to find salivary microRNAs (miRNAs) reflecting periodontal condition in chronic periodontitis. One hundred and twenty chronic periodontitis patients (mean age, 68.4 years) participated in the study, from whom unstimulated whole saliva was collected. A multiphase study was conducted to explore salivary miRNAs as biomarkers of periodontitis. At first, a polymerase chain reaction (PCR) array was performed to compare salivary miRNAs profiles in no and mild (no/mild) and severe periodontitis patients. Next, the relative expression of salivary miRNAs on individual samples was assessed by real-time reverse transcription-PCR. The numbers (%) of patients were 26 (21.6%, no/mild), 58 (48.3%, moderate) and 36 (30.0%, severe), respectively. Among 84 miRNAs, only the relative expression of hsa-miR-381-3p in the severe periodontitis group was significantly higher than that of the no/mild periodontitis group (*p* < 0.05). Among the 120 patients, there was also a significant correlation between the relative expression of hsa-miR-381-3p and the mean probing pocket depth (PPD) (r = 0.181, *p* < 0.05). Salivary hsa-miR-381-3p was correlated with periodontitis condition in chronic periodontitis patients.

## 1. Introduction

Periodontitis is a chronic inflammation which causes the destruction of gingival connective tissue, periodontal ligament, cementum and alveolar bone loss. Periodontitis is classified as no periodontitis, mild periodontitis, moderate periodontitis or severe periodontitis, according to the degree of disease progression [1,2]. The risk of tooth loss caused by periodontitis varies according to disease progression [3]. Therefore, development of examination methods for periodontal condition is important for estimating the risk of tooth loss.

Traditionally, periodontitis severity is evaluated by measuring probing pocket depth (PPD) and clinical attachment levels (CAL) [1]. However, measuring PPD and CAL is a technically demanding process that requires a high degree of skill. In addition, these parameters do not reflect present periodontitis activity, but simply assess the cumulative effects of periodontal tissue destruction. A new rational diagnosis of periodontitis condition would have concomitant patient benefits because the paucity of evidence-based knowledge of disease severity in individual periodontitis patients may lead to unintentional clinical mismanagement.

Recent studies have reported that measuring salivary mediators can be useful in determining periodontal condition [4,5]. For example, cytokines [6], lactate dehydrogenase [7,8,9,10,11], lactoferrin [12,13,14] and 8-hydroxydeoxyguanosine [15,16,17] are known as possible salivary biomarkers for periodontal condition. Salivary mediators associated with periodontal condition may help in the development of novel diagnostics for periodontitis.

miRNAs (micro RNAs) are single strained non-coding small RNAs (~22-nucleotide) which regulate post-transcriptional repression of target messenger RNAs (mRNAs) [18]. MiRNAs exist in several body fluids, including saliva. Recent studies have shown that periodontitis can modulate the periodontal tissue levels of miRNAs [19,20], which may also change miRNA profile in saliva [21]. However, the relationship between salivary miRNAs profile and periodontal condition remains obscure.

In the present work, we hypothesized that salivary miRNA profiles vary according to the periodontal condition. Therefore, the aim of this cross-sectional pilot study was to investigate the relationship between salivary miRNAs and periodontal condition in chronic periodontitis patients.

## 2. Results

### 2.1. Characteristics of Patients

One hundred and twenty patients (mean age, 68.4) were recruited in this study and were classified according to periodontal severity (see Methods). The number of participants of the no/mild periodontitis group, the moderate periodontitis group, and the severe periodontitis group was 26, 58, and 36 respectively (Table 1). We evaluated the characteristics of the patients, including age, gender, number of teeth present, mean PPD, mean CAL, bleeding on probing (BOP) (%), plaque control record (%), smoking status, presence of diabetes mellitus, and frequency of tooth brushing per day. In terms of patient characteristics, except age, mean PPD, mean CAL, BOP and number of teeth, significant differences were not recognized between the groups.

### 2.2. Selection of Candidate miRNAs Reflecting Periodontitis

To screen candidate miRNAs reflecting periodontitis, Inflammatory Response and an Autoimmunity miScript miRNAs PCR Array (SA Biosciences, Frederick, MD) using pooled sample (no/mild periodontitis group vs severe periodontitis group, *n* = 3/group) were conducted. Among 84 miRNAs, there was only a significant difference in hsa-miR-381-3p between the no/mild periodontitis group and the severe periodontitis group (*p* = 0.015). Furthermore, three miRNAs (hsa-miR381-3p, hsa-miR-543, and hsa-miR-144-3p) were upregulated (fold change > 2) in the severe periodontitis group, as compared with the no/mild periodontitis group (Table 2, Figure S1). One miRNA (hsa-30b-5p) was down-regulated (fold change < 0.5) in the severe periodontitis group, as compared with the no/mild periodontitis group. From these data, hsa-miR-381-3p, hsa-miR-543, hsa-miR-144-3p, and hsa-miR-30b-5p were selected for further analysis.

### 2.3. Evaluating Validation of Selected miRNAs

**Figure 1:**
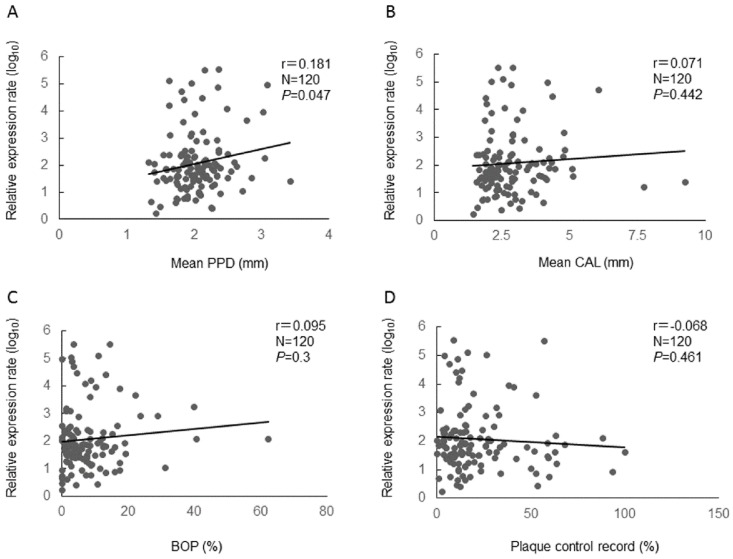
Relative expression of hsa-miR-381-3p and clinical parameters (**A**: mean PPD, **B**: mean CAL, **C**: BOP, and **D**: Plaque control record) using Pearson’s correlation coefficients between. N: number, r: Pearson’s correlation coefficients, *p*: *p* value. The relative expression of hsa-miR-381-3p was normalized by log10 transformation for the analysis.

We selected hsa-miR-381-3p, hsa-miR-543, hsa-miR-144-3p, and hsa-miR-30b-5p and performed the quantitative real-time PCR assays by inventoried TaqMan miRNA assays (Applied Biosystems, Foster City, CA, USA) for all participants (Figure S2). There were no significant differences in miRNAs expression among three groups. Next, we evaluated the relationships between the relative expressions of the miRNAs and clinical parameters (mean PPD, mean CAL, BOP, and Plaque control record) by Pearson’s correlation test. There was a significant positive mild correlation between the relative expression of hsa-miR-381-3p and mean PPD (r = 0.181, *p* = 0.047) (Figure 1). In addition, we examined the relationship between hsa-miR-381-3p and mean PPD by the stepwise method of multiple linear regression analysis. It was also shown that the relative expression of hsa-miR-381-3p was significantly correlated with mean PPD (*p* = 0.049) (Table 3) by the multiple linear regression analysis. For the other miRNAs, there was no significant correlation between the relative expressions of the miRNAs and the clinical parameters.

### 2.4. Bioinformatics

With regard to hsa-miR-381-3p, the target genes were strongly associated with pathways for cancer, focal adhesion, Wnt signaling, and MAPK (mitogen-activated protein kinase) signaling (Table 4).

## 3. Discussion

In this study, we hypothesized that miRNAs reflecting periodontal condition exist in saliva. In PCR array analysis, we selected hsa-miR-381-3p as a biomarker of periodontitis severity. In addition, we confirmed that relative expression of hsa-miR-381-3p was correlated with mean PPD by multiple linear regression analysis. The results indicate that salivary hsa-miR-381-3p may reflect periodontal condition in chronic periodontitis patients. This is consistent with previous results, revealing that the miR-381 in blood samples was one of the most significantly related miRNAs with chronic periodontitis [22]. However, the effect sizes (Pearson’s correlation coefficients, 0.071–0.181) were small. It is possible that biological factors which we could not evaluate in this study may affect clinical parameters and obscure the correlation with hsa-miR-381-3p.

There is little research carried out on the potential role of miR-381 in the innate immune response. However, a previous study found that treatment with LPS (lipopolysaccharide) elevated miR-381 expression in A549 cells (immortalized cells derived from human adenocarcinomas’ alveolar basal epithelial cells isolated from the lungs) in a time- and dose-dependent manner [23]. LPS is one of bacterial pathogens and immune responses to LPS play a crucial role in the pathogenesis of periodontitis [24]. In this study, there was no statistical correlation between hsa-miR-381-3p and plaque control records, suggesting that the expression of hsa-miR-381-3p did not directly reflect supragingival bacterial pathogens. On the other hand, the up-regulated expression of hsa-miR-381-3p in our observations may be associated with the increased immune responses to subgingival bacterial pathogens such as LPS. In addition, LPS is related to interferons in innate immune response, and there is a correlation between interferon-gamma in the crevicular fluid and periodontitis [25]. Interferon-gamma may correlate with the expression of hsa-miR-381-3p. Therefore, we need to carry out further study in the future about the relationship between miRNA expression and subgingival periodontopathic bacteria in immune response.

Studies have shown the relationship between periodontitis and miRNA profiles in periodontal tissue. For example, with regard to miR-181b, miR-19b, miR-23a, miR-30a, miR-let7a and miR-301a, miRNAs levels in periodontal tissue of chronic periodontitis patients are higher than those of healthy controls [26]. Another study reported that hsa-miR-150, hsa-miR-223 and hsa-miR-200b were overexpressed and hsa-miR-379, hsa-miR-199a-5p and hsa-miR-214 were underexpressed in inflamed gingival tissues [27]. Furthermore, it has been demonstrated that gingival tissue levels of miR-146a are correlated with periodontal parameters [28]. These observations show that periodontitis is able to modulate the expression of miRNAs in the periodontal tissue. In the present study, we found that salivary expression of hsa-miR-381-3p is correlated with PPD in chronic periodontitis patients. Periodontitis may up-regulate the expression of hsa-miR-381-3p in periodontal tissue, and such a condition would contribute to an increase of hsa-miR-381-3p in saliva. However, in the present study, relative expression of hsa-miR-381-3p was correlated with mean PPD, but not with mean CAL. This indicates that hsa-miR-381-3p reflected periodontal status rather than the present periodontal disease. Because the expression of hsa-miR-381-3p was correlated with only periodontal status, it is possible that the changes in hsa-miR-381-3p expression was not found from the periodontal disease tissues in the other studies [19,27]. In addition, hsa-miR-381-3p might be derived not only from gingival tissues directly, but also from other tissues affected by periodontitis. Further studies are needed to clarify if this is the case.

Bioinformatics analysis showed that the target genes of hsa-miR-381-3p correlated with MAPK signaling pathway. MAPK signaling is closely related to the pathology of periodontitis [29]. Therefore, it is possible that the modulated expression of hsa-miR-381-3p in saliva has an influence on periodontal condition through the MAPK signaling pathway. The expression of hsa-miR-381-3p in saliva not only reflects periodontal condition, but may also affect the periodontal tissue. However, because this study was cross-sectional, further studies are needed to clarify this point.

A clinical study demonstrated that saliva could be a feasible diagnostics tool of tongue squamous cell carcinoma and that miR-139-5p could be a potential biomarker of early tongue squamous cell carcinoma detection [30]. The other study also revealed that miR-4484 was significantly upregulated in the salivary exosomes of patients with oral lichen planus [31]. Furthermore, in the present study, there was a positive correlation between hsa-miR-381-3p and periodontal condition. The previous and present results support the notion that salivary miRNAs are novel biomarkers for oral diseases.

In general, periodontal condition was evaluated using X-ray images, and clinical parameters such as PPD, CAL, mobility and BOP. However, because these methods need some experiences and physical burden or pain for periodontitis patients, diagnosis by a dentist is necessary. On the other hand, saliva is one of the least invasive and easiest body fluids to collect. Therefore, anyone can perform salivary tests. Periodontitis often progresses without subjective symptoms. It may therefore contribute to the screening of periodontitis progression if we establish a system that can indicate severe periodontitis by saliva test at home.

There are some limitations in this study. First, in PCR array analysis, we analyzed 84 miRNAs expressions related to the inflammatory response and autoimmunity. However, it is possible that other miRNAs are associated with periodontitis. Second, external validity is limited because all of the patients were recruited from Okayama University Hospital. Third, we used PCR array to select candidate miRNAs reflecting periodontitis. However, because the human genome may encode over 1000 miRNAs, next generation sequencing is recommended to investigate the whole transcriptome. Fourth, this was a cross-sectional pilot study. It is necessary to improve the reliability of this study by researching the longitudinal relationship between hsa-miR-381-3p and periodontal condition.

## 4. Materials and Methods

### 4.1. Experimental Design and Participants’ Recruitment

**Figure 2:**
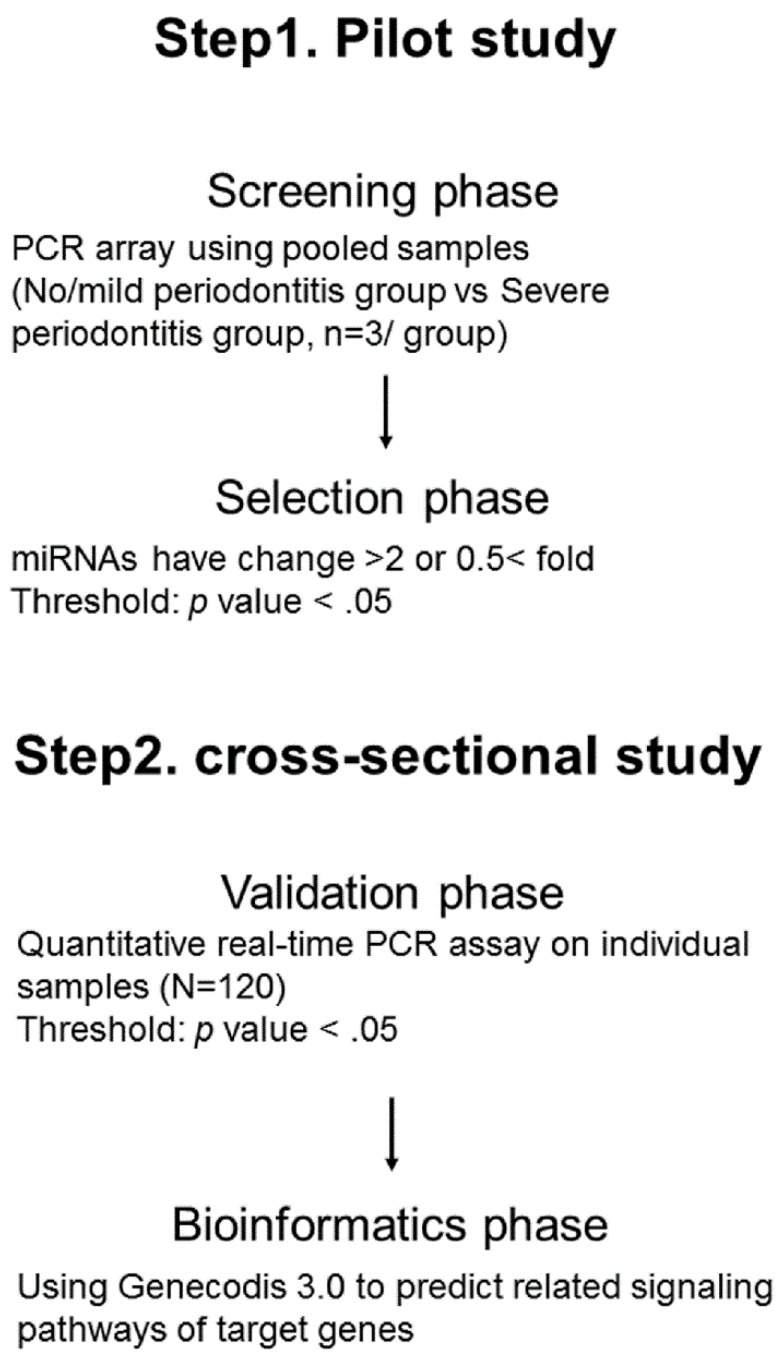
An overview of the experimental design.

This study was carried out according to the Declaration of Helsinki (World Medical Association, 2002) and was admitted by the Ethics Committee of Okayama University Hospital (approval number: 1603-002). This study was designed as multiphase study (screening phase and selection phase, validation phase, and bioinformatics phase) to identify salivary miRNAs as biomarkers of periodontitis (Figure 2). In the screening phase and the selection phase (a pilot study), a comparison of salivary miRNAs profiles with no and mild (no/mild) and severe periodontitis patients was done to select candidate miRNAs reflecting periodontitis. In the next two phases (a cross-sectional study), the validation of selected salivary miRNAs in a pilot study was confirmed using the individual samples. After obtaining written informed consent, we recruited 120 patients (median age, 68.4 years) for participants from the Department of Preventive Dentistry, Okayama University Hospital. Participant recruitment was carried out from July 2016 to October 2016. This sample size was based on the number of patients that we were able to recruit during a 4-month period. All participants had been diagnosed with chronic periodontitis [32], and they did not have oral pain, oral disorders or dental treatments other than supportive periodontal therapy. Exclusion criteria were as follows; pregnancy, less than 40 years of age, use of anti-inflammatory drug, or the onset of acute inflammation of periodontal tissue within the 3-month period before the oral examinations.

### 4.2. Oral Examination

PPD, CAL and BOP were examined. Measurement points of PPD and CAL were six sites on all teeth (mesio-buccal, mid-buccal, disto-buccal, mesio-lingual, mid-lingual and disto-lingual). We measured PPD and CAL using a dental probe (Hu-Friedy, Chicago, IL, USA). Bleeding sites on probing (25 g probing force) were counted and the percentage of bleeding sites was calculated. Plaque was stained with erythrosine. At four sites (mesial, distal, buccal and lingual) around each tooth, we recorded sites where plaque was present and calculated percentage of plaque presence sites [33]. Six trained and calibrated dentists (T. T., D. E., T. A., T. M., H. M. and T. Y.) carried out all clinical procedures. The non-parametric kappa test and intra-class correlation was used for the data analysis. The k coefficients for intra- and inter-examiner and intra-class correlation coefficients were >0.8. After oral examination, we classified periodontitis severity (no/mild periodontitis, moderate periodontitis, or severe periodontitis) with accordance to the joint Center for Disease Control/American Association of Periodontology working group [1]. This section is not mandatory, but can be added to the manuscript if the discussion is unusually long or complex.

### 4.3. Questionnaire

Participants answered a questionnaire. Questionnaire items covered smoking status, body mass index (BMI) (weight in kilograms per square of height in meters), presence of diabetes mellitus, and frequency of tooth brushing per day.

### 4.4. Saliva Collection and RNA Extraction

Unstimulated whole saliva (1–2 mL) was collected as reported previously [34]. Saliva was collected from 7:00 AM to 12:00 on the same day as oral examination. In this study, any inhibitors or preservatives were not added to the saliva. After saliva was collected, we removed cells and debris in saliva by centrifugation (10 min, 2000 g, room temperature). Supernatant was isolated and stored at 4 °C for up to 6 h, then it was stored at −80 °C until it was used. We used total exosome isolation reagent (Invitrogen, Carlsbad, CA, USA) to extract Exosome in saliva samples (300 μL). A total exosome RNA and protein isolation kits (Invitrogen, Carlsbad, CA, USA) [34] were used to isolate total RNA, containing miRNAs from salivary exosome samples. Agilent 2100 Bioanalyzer (Agilent Technologies, Santa Clara, CA, USA) was used to confirm quality of total RNA, which contained <500 nucleotides and no/little 18S and 28S ribosomal RNA.

### 4.5. Selection of Candidate miRNAs Reflecting Periodontitis

In the screening phase, PCR array analysis was performed in triplicate. The severe periodontitis patients and the no/mild periodontitis patients were divided into three subgroups respectively (no/mild 1 [N = 9], no/mild 2 [N = 9], no/mild 3 [N = 8], severe 1 [N = 12], severe 2 [N = 12], and severe 3 [N = 12]). The Inflammatory Response and Autoimmunity miScript miRNAs PCR Array (SA Biosciences, Frederick, MD) was conducted. This array had 84 miRNA assays, which includes candidate housekeeping small RNA (SNORD61, SNORD68, SNORD95, SNORD96A and RNU6-6p). Reverse transcription was conducted using the miScript II RT kit (QIAGEN, Hilden, Germany) for reverse transcription. The data was analyzed using an online analysis tool (SA Biosciences; http://pcrdataanalysis.sabiosciences.com/mirna). We used the 2^−ΔΔCt^ method to analyze the relative quantification of miRNAs [35]. In addition, we made a heat map to visualize the results of one-way hierarchical clustering of miRNAs in the six subgroups (SA Biosciences; http://pcrdataanalysis.sabiosciences.com/mirna) from the Ct values of the array. In the selection phase, we selected candidate miRNAs that showed statistically significant (*p* < 0.05) fold changes (<0.5 or >2).

### 4.6. Evaluating Validation of Selected miRNAs

In the validation phase, reverse transcription polymerase chain reaction (RT-PCR) analysis on individual samples (N = 120) was conducted. TaqMan microRNA Assays (Life Technologies (Thermo Fisher Scientific, Carlsbad, CA, USA) and the Mx3000P Real-time QPCR System (Agilent Technologies, Santa Clara, CA, USA) were used [36,37]. The thermal cycler was set at 50 cycles. We included data with a threshold cycle (Ct) value of <50 in each analysis. The relative expression rates of each miRNA were calculated using the 2^−ΔΔCt^ method [35]. Internal control miRNA was U6 snRNA (Figure S3). We normalized the relative expression of candidate miRNA by log10 transformation for analysis.

### 4.7. Bioinformatics

In the bioinformatics phase, we predicted target genes of candidate miRNAs using miRWalk [38]. We used TargetScan, miRanda, and miRWalk for multiple bioinformatics analysis in miRWalk. We included at least two databases to predict target genes. We predicted related signaling pathways of these predicted target genes using genedodis 3.0 [39,40,41].

### 4.8. Statistical Analysis

SPSS statistics version 22.0 (IBM Japan, Tokyo, Japan) was used for statistical analysis. We used a Student’s *t*-test to compare the no/mild periodontitis group with the severe periodontitis group for PCR array. The data are presented as mean values ± standard deviation (SD). The differences in miRNA expressions among three groups were analyzed by one-way ANOVA followed by the Tukey’s method. A Pearson’s correlation test was carried out to analyze the relationships between candidate miRNAs and clinical parameters (mean PPD, mean CAL, BOP, and Plaque control record). We also examined the relationship between candidate miRNAs and significantly correlated variables by the stepwise method of multiple linear regression analysis. We considered *p* < 0.05 as significant differences.

## 5. Conclusions

The relative expression of salivary hsa-381-3p was correlated with periodontal condition in chronic periodontitis patients within the limitation. Measuring salivary hsa-381-3p may be a useful new biomarker for periodontitis.

## Figures and Tables

**Table 1:** Characteristics of the participants (*n* [%] or mean [SD]).

Variables	Categories	Total (N = 120)	Severity of Periodontitis			
			No/Mild	Moderate	Severe	
			(N = 26)	(N = 58)	(N = 36)	*p* Value
Age (years)		68.4 (10.2)	63.3 (13.9)	68.6 (8.7)	71.7 (7.9)	0.005 *
Gender	Male	38 (31.7)	6 (23.1)	18 (31.0)	14 (38.9)	0.414 ^†^
Number of teeth present		23.6 (5.0)	25.5 (3.7)	24.3 (4.3)	21.0 (6.0)	<0.001 *
Mean PPD (mm)		2.0 (0.4)	1.9 (0.2)	2.0 (0.3)	2.3 (0.5)	<0.001 *
Mean CAL (mm)		2.8 (1.2)	1.9 (0.2)	2.6 (0.7)	3.9 (1.4)	<0.001 *
BOP (%)		7.7 (9.0)	6.9 (9.2)	5.8 (4.9)	11.3 (12.7)	0.015 *
Plaque control record (%)		22.2 (20.3)	18.1 (14.7)	24.0 (20.3)	22.3 (24.0)	0.486 *
Smoking status	Current	6 (5.0)	0 (0)	2 (3.4)	4 (11.1)	0.109 ^†^
BMI		22.5 (3.0)	22.0 (2.7)	22.2 (2.7)	23.3 (3.3)	0.162 *
Diabetes mellitus	Present	12 (10.0)	2 (7.7)	8 (13.8)	3 (5.6)	0.392 ^†^
Frequency of toothbrushing/day		2.5 (0.7)	2.6 (0.6)	2.3 (0.7)	2.7 (0.8)	0.052 *

* one-way analysis of variance. ^†^ Chi-square test. PPD, probing pocket depth; CAL, clinical attachment level; BOP, bleeding on probing; BMI, body mass index.

**Table 2:** List of the differentially expressed miRNAs between severe and no/mild periodontitis (Fold Change >2 or < 0.5).

miRNAs	Average Ct		Average ⊿Ct		Average 2^-⊿Ct		Fold Change	*p* Value
	No/Mild	Severe	No/Mild	Severe	no/Mild	Severe		
hsa-miR-381-3p	34.88	33.05	2.35	0.49	0.20	0.71	3.63	0.01
hsa-miR-543	34.89	33.48	2.36	0.92	0.20	0.53	2.70	0.12
hsa-miR-144-3p	33.92	32.84	1.39	0.28	0.38	0.82	2.16	0.51
hsa-miR-30b-5p	30.05	31.32	−2.48	−1.24	5.57	2.36	0.42	0.14
housekeeping small RNA	32.53	32.56						

Fold-change values greater than one indicate up-regulation and less than one indicate down-regulation. Average ⊿Ct = Average Ct (miRNA) − Average ⊿Ct (housekeeping small RNA). Fold change = 2^ (-Average⊿Ct (severe))/2^ (-Average⊿Ct (no/mild)). Average Ct values of housekeeping small RNAs mean average Ct of SNORD61, SNORD68, SNORD95, SNORD96A and RNU6-6p. The *p* value was calculated by the Student’s t-test.

**Table 3:** Multiple linear regression analysis with mean PPD as the dependent variable (N = 120).

Variables	B (95%CI)		β	*p* Value	VIF
Intercept	1.917	(1.760, 2.073)	-	<0.001	-
BOP	0.014	(0.008, 0.021)	0.353	<0.001	1.036
Gender	−0.148	(−0.279, −0.017)	−0.187	0.027	1.036
hsa-miR-381-3p	0.054	(0.010, 0.123)	0.166	0.049	1.022

The candidate variables for the model were age, gender, BOP (%), Plaque control record (%), number of teeth present, and hsa-miR-381-3p. We selected candidate valuables which have significantly correlated with mean PPD in Pearson’s correlation analysis (*p* < 0.05). The final model was constructed based on the maximum adjusted R-squared and VIF less than 10. The F-statistic was 10.392 (*p* < 0.001), the R-squared was 0.212, and the adjusted R-squared was 0.191. N: number. CI: confidence interval. B: unstandardized regression coefficient. β: standardized coefficient. VIF: variance inflation factor. PPD: probing pocket depth. BOP: bleeding on probing.

**Table 4:** Pathway analysis of target genes for hsa-miR-381-3p.

Pathway	Target Genes	*p*-Value
Pathways in cancer	*DVL2*, *PTCH1*, *SOS1*, *EGLN3*, *MET*, *LEF1*, *MAPK1*, *STAT1*, *KITLG*, *PIAS2*, *DCC*, *FGF1*, *FZD6*, *KIT*, *FOXO1*, *PRKCB*, *GLI3*, *NRAS*, *NFKBIA*, *SMAD2*, *BIRC3*, *AKT3*, *BRAF*, *MAPK10*, *PRKCA*, *CRK*, *LAMC1*, *MAPK8*, *COL4A1*, *TRAF6*, *FZD4*, *VHL*, *MITF*, *ITGAV*, *WNT5A*, *RAC1*, *FZD3*, *PTK2*, *FGF7*, *PIK3CG*, *TP53*, *VEGFA*, *HSP90AB1*, *FGF12*, *HDAC2*, *CBLB*, *PTGS2*, *FGFR2*	1.02 × 10^−12^
Focal adhesion	*SOS1*, *MET*, *MAPK1*, *PDGFC*, *TLN2*, *ITGA4*, *PRKCB*, *ACTG1*, *ROCK2*, *PDGFD*, *CCND2*, *MYL12B*, *BIRC3*, *AKT3*, *ITGA8*, *BRAF*, *PARVA*, *MAPK10*, *PRKCA*, *CRK*, *LAMC1*, *MAPK8*, *COL4A1*, *MYLK3*, *ITGAV*, *RAC1*, *PTK2*, *FYN*, *PIK3CG*, *ITGB8*, *VEGFA*, *COL11A1*	1.48 × 10^−9^
Wnt signaling pathway	*DVL2*, *SIAH1*, *LEF1*, *MAP3K7*, *FZD6*, *PRKCB*, *ROCK2*, *CCND2*, *PPP2R5A*, *SMAD2*, *CXXC4*, *MAPK10*, *PRKCA*, *PPP2R5E*, *MAPK8*, *SFRP2*, *LRP6*, *FZD4*, *PRKACG*, *WNT5A*, *NFATC2*, *RAC1*, *FZD3*, *NFAT5*, *CAMK2G*, *TP53*, *PRKACB*	2.90 × 10^−9^
MAPK signaling pathway	*NTRK2*, *CACNA2D1*, *SOS1*, *MAPK1*, *MAP3K7*, *FGF1*, *CACNA1C*, *CACNA1E*, *PRKCB*, *IL1R1*, *NRAS*, *DDIT3*, *AKT3*, *BRAF*, *ELK4*, *MAPK10*, *TAOK1*, *PRKCA*, *CRK*, *MAPK8*, *TRAF6*, *PRKACG*, *MAPK14*, *RRAS2*, *NFATC2*, *RAC1*, *FGF7*, *RPS6KA3*, *ARRB1*, *TP53*, *FGF12*, *CACNB2*, *PRKACB*, *FGFR2*	6.83 × 10^−8^
Regulation of actin cytoskeleton	*ARHGEF7*, *SOS1*, *MAPK1*, *FGF1*, *PDGFC*, *ITGA4*, *ACTG1*, *ROCK2*, *PDGFD*, *NRAS*, *SSH2*, *MYH10*, *CFL2*, *MYL12B*, *NCKAP1*, *ITGA8*, *MYH9*, *BRAF*, *GNA13*, *CRK*, *MYLK3*, *ITGAV*, *RRAS2*, *RAC1*, *PTK2*, *FGF7*, *PIK3CG*, *ITGB8*, *FGF12*, *FGFR2*	6.94 × 10^−8^
Insulin signaling pathway	*SOS1*, *MAPK1*, *EIF4E*, *FOXO1*, *NRAS*, *PRKAR2B*, *RPS6KB1*, *AKT3*, *BRAF*, *PRKAR2A*, *SOCS4*, *MAPK10*, *CRK*, *MAPK8*, *PRKACG*, *PRKAG3*, *RHOQ*, *PRKAA2*, *PIK3CG*, *SORBS1*, *PHKG2*, *PRKACB*, *CBLB*	1.01 × 10^−7^
Ubiquitin mediated proteolysis	*ANAPC10*, *UBE2C*, *SIAH1*, *HERC3*, *PIAS2*, *UBR5*, *CUL4B*, *CDC23*, *HERC4*, *ITCH*, *ANAPC13*, *UBE3A*, *BIRC3*, *UBE2W*, *UBE2G1*, *TRIM32*, *NEDD4*, *MID1*, *TRAF6*, *SYVN1*, *VHL*, *UBE2E2*, *CBLB*	1.17 × 10^−7^
ErbB signaling pathway	*SOS1*, *MAPK1*, *PRKCB*, *NRAS*, *ABL2*, *RPS6KB1*, *NCK1*, *AKT3*, *BRAF*, *HBEGF*, *MAPK10*, *PRKCA*, *CRK*, *MAPK8*, *PTK2*, *CAMK2G*, *PIK3CG*, *CBLB*	1.86 × 10^−7^
Long-term potentiation	*MAPK1*, *CAMK4*, *CACNA1C*, *PRKCB*, *NRAS*, *GRM5*, *BRAF*, *PRKCA*, *GRM1*, *ITPR2*, *GNAQ*, *PRKACG*, *GRIN2A*, *RPS6KA3*, *CAMK2G*, *PRKACB*	1.97 × 10^−7^
Axon guidance	*SEMA3C*, *CXCR4*, *MET*, *MAPK1*, *DCC*, *ROCK2*, *NRAS*, *CFL2*, *ABLIM3*, *NCK1*, *NTN4*, *ABLIM1*, *NFATC2*, *RAC1*, *PTK2*, *NFAT5*, *UNC5D*, *FYN*, *EFNB2*, *EPHA3*, *SRGAP2*	6.88 × 10^−7^

Multiple bioinformatics databases including TargetScan, miRanda, and miRWalk were used. Predicted targets were included in three databases. We used Genecodis 3.0 to predict related signaling pathways of these predicted target genes.

## Supplementary Materials

The following are available online, Figure S1: Scatterplot of the results of miRNA PCR array analysis, Figure S2: Relative expressions of miRNAs (A: hsa-miR-381-3p, B: hsa-miR-543, C: hsa-miR-144-3p, and D: hsa-miR-30b-5p) among three groups using quantitative real-time PCR assays, Figure S3: Ct values of U6.
