# Supplementary material for: Detection of Salivary miRNAs Reflecting Chronic Periodontitis: A Pilot Study
Source: Molecules. 2019 Mar 15;24(6):1034. doi: 10.3390/molecules24061034 (PMC6470766; doi:10.3390/molecules24061034)
Supplement: Supplementary file 1 [file molecules-24-01034-s001.zip › molecules-449473 supple 1/Figure S1.pdf]

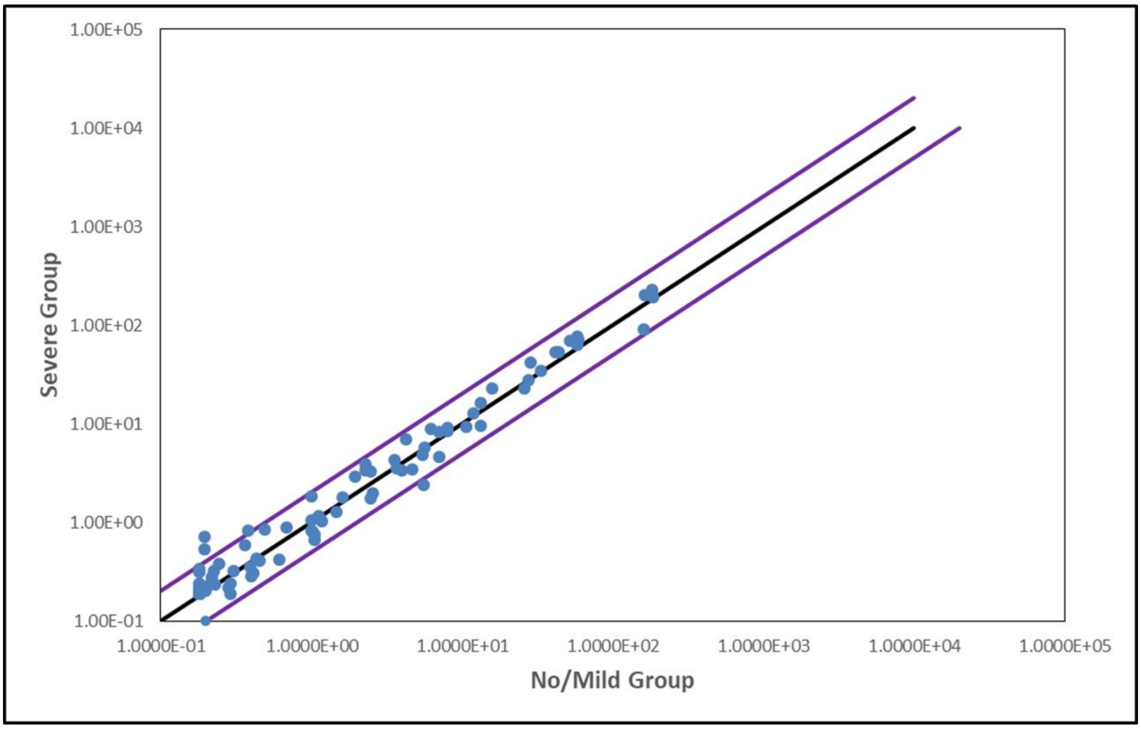

Figure S1. Scatterplot of the results of miRNA PCR array analysis.

This plot shows the hybridization signal patterns of miRNAs for the No/Mild and the Severe groups. The expression of miRNAs above the top purple line or below the down purple line shows the difference with fold change >2.0 between the Severe and the No/Mild samples.
