# Supplementary material for: Detection of Salivary miRNAs Reflecting Chronic Periodontitis: A Pilot Study
Source: Molecules. 2019 Mar 15;24(6):1034. doi: 10.3390/molecules24061034 (PMC6470766; doi:10.3390/molecules24061034)
Supplement: Supplementary file 1 [file molecules-24-01034-s001.zip › molecules-449473 supple 1/Figure S2.pdf]

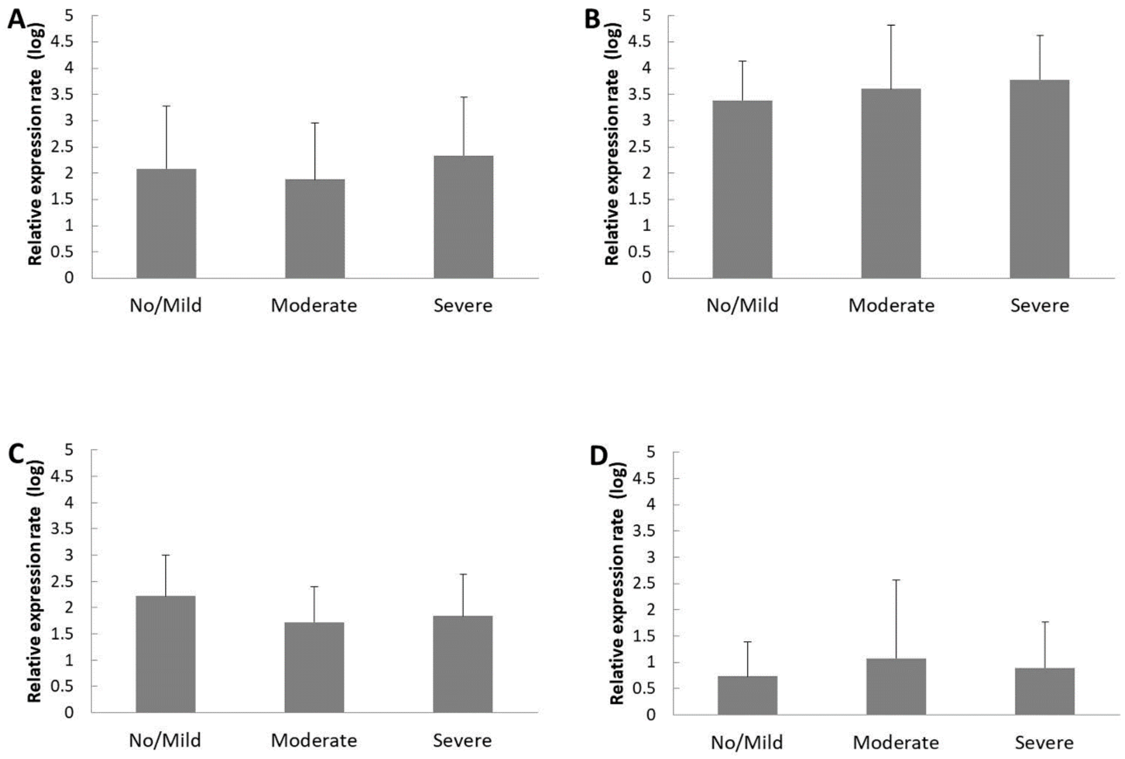

Figure S2. Relative expressions of miRNAs (A: hsa-miR-381-3p, B: hsa-miR-543, C: hsa-miR-144-3p, and D: hsa-miR-30b-5p) among three groups using quantitative real-time PCR assays.

One way ANOVA followed by Tukey’s method was used.

Bars represent mean ± SD of each periodontitis groups.

The relative expressions of miRNAs were normalized by log10 transformation for the analysis.
