# Supplementary figures and images for: Detection of Salivary miRNAs Reflecting Chronic Periodontitis: A Pilot Study
Source: Molecules. 2019 Mar 15;24(6):1034. doi: 10.3390/molecules24061034 (PMC6470766; doi:10.3390/molecules24061034)

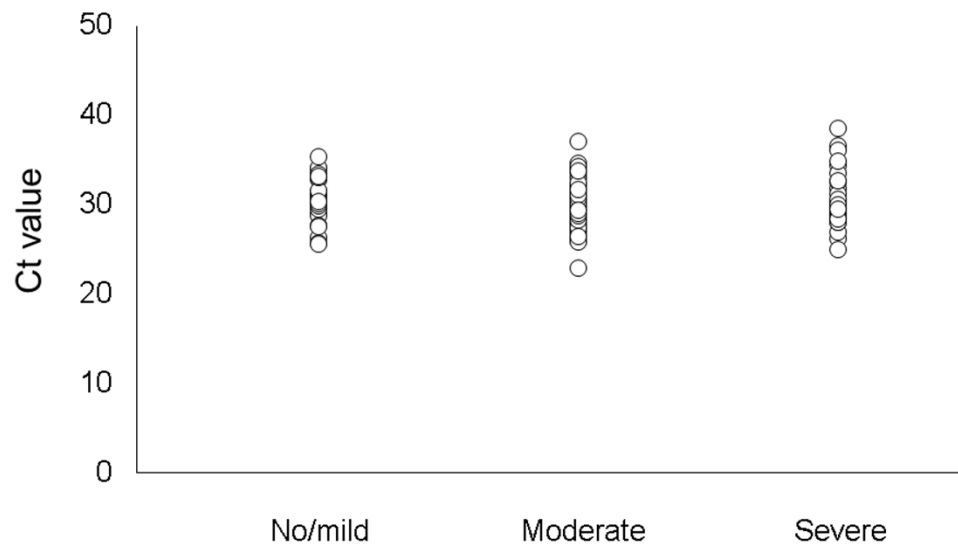

**Figure S3.** Ct values of U6.

Supplement: Supplementary file 1 [file molecules-24-01034-s001.zip › molecules-449473 supple 1/Figure S3.pdf]
