# Supplementary material for: Detection of Salivary miRNAs Reflecting Chronic Periodontitis: A Pilot Study
Source: Molecules. 2019 Mar 15;24(6):1034. doi: 10.3390/molecules24061034 (PMC6470766; doi:10.3390/molecules24061034)
Supplement: Supplementary file 1 [file response to reviewer.pdf › molecules-449473 supple 1/response to reviewer.pdf]

Our responses to reviewers

Reviewer: 1

Comments to the Author

In this original paper Fujimori et al conducted a pilot study to detect possible microRNAs that could be useful to diagnose and predict chronic periodontitis. The authors first perform PCR array and find miR-381-3p as a suitable candidate. Second, the authors validated by qRT-PCR the array data.
The paper is interesting and data is presented structured and logical, but there are some pitfalls that raise my concern:

qRT-PCR data is missing – add a Figure were you show the expression variation between the 3 groups (mild vs moderate vs severe). Was the array data confirmed? qRT-PCR must be performed for miR-381-3p, miR-543, miR-144 and miR-30b-5p. If the array data cannot be confirmed this research cannot be published.

Our response:
Thank you for your comments. We have confirmed the array data. We have performed qRT-PCR for miR-543, miR-144 and miR-30b-5p following the reviewer's suggestion. There were no significant differences in these expressions between the three groups the array data. We have revised the manuscript (page 4, L108-110) and added the Figure S2 as the supplemental figure.

How stable was the normalizer between the patient groups in qRT-PCR? Add a graphic in supplementary data were you show the stability of U6 between groups. Also it is recommended to use two different normalizers.

Our response:
Thank you for your comments. The expression of U6 was stable and there were no significant defferences in the expression of U6 between the three groups. We have added the Figure S3 as the supplemental figure.
U6 has been commonly used for comparison of miRNA expression in other studies. Thus, we concluded that it was enough to use U6 for normalizer.

50 cycles threshold is too much, recommended is 40.

Our response:
Thank you for your comment. However, we needed over 40 cycles to detect miRNA expression in all samples. Therefore, we set 50 cycles threshold.

Add figure were you depict the array results.

Our response:
Thank you for your comment. We have added the Figure S1 as the supplemental figure to show the array results.

No research on mechanism performed except some simplistic bioinformatics data prediction. Would be also interesting to perform some common target predictions with the other 3 miRNAs detected by array. Also some IPA analysis would add more power to your research.

Our response:
Thank you for your comments. As the reviewer suggested, it would be interesting to perform some common target predictions with the other three miRNAs detected by array and IPA analyses. However, we focused hsa-miR-381-3p because there were no significant differences in the expression of the other three miRNAs. The suggestion will be included in a future research.

Reviewer: 2

Comments to the Author

The manuscript is nicely presented with a clear objective, outcome and conclusion.
In your introduction section please include the reference for the new classification scheme for periodontal disease...
This is the full reference:
Caton JG, Armitage G, Berglundh T, Chapple ILC, Jepsen S, Kornman KS, Mealey BL, Papapanou PN, Sanz M, Tonetti MS (2018) A new classification scheme for periodontolal and peri-implant diseases and conditions – Introduction and key changes from the 1999 classification. J Clin Periodontol 45, 45(20), S1-S8. doi: 10.1111/jcpe.12935

Our response:
Thank you for your comments. We have added the reference and revised the manuscript following the reviewer's suggestion (page 1, L37).

Results page 2/12 line 63, please add according to between classified and periodontal severity.

Our response:
Thank you for your comment. We have inserted the words, "according to" between "classified" and "periodontal severity" following the reviewer's suggestion (page 2, L63).

Discussion page 6/12 line 113. There is little, not are....delete which.. so the line should read There is little research carried out....

Our response:
Thank you for your comments. We have changed the word following the reviewer's suggestion (page 7, L146).

line 134 page 7/12 contribute to an increase of ...

Our response:
Thank you for your comments. We have changed the word following the reviewer's suggestion (page 8, L167).

Line 141 Further studies are needed. Can you extend this by adding ..are needed to clarify if this is the case.

Our response:

Thank you for your comments. We have changed the words following the reviewer's suggestion (page 8, L174).
